# Intraventricular Neurocysticercosis: Experience and Long-Term Outcome from a Tertiary Referral Center in the United States

**DOI:** 10.4269/ajtmh.18-0085

**Published:** 2018-04-23

**Authors:** Theodore E. Nash, JeanAnne M. Ware, Siddhartha Mahanty

**Affiliations:** Laboratory of Parasitic Diseases, National Institutes of Allergy and Infectious Diseases, National Institutes of Health, Bethesda, Maryland

## Abstract

Ventricular involvement in neurocysticercosis (NCC), a common serious manifestation of NCC, has distinct clinical presentations, complications, and treatments primarily because of partial or complete obstruction of the cerebrospinal fluid (CSF) flow by *Taenia solium* cysts. We review the clinical course, treatments, and long-term outcomes in 23 of 121 (19.0%) total NCC patients with ventricular cysts referred to the National Institutes of Health from 1985 to the October 2017. Patients had a median age of 31.8 (range: 22.4–52.6 years), were 60.9% male, diagnosed a median of 6.5 years (range: 0.17–16 years) after immigration, and were followed for a median of 3.6 years (range: 0.1–30.5 years). Other forms and manifestations of NCC were present in 73.9% (17/23). The fourth ventricle was involved in a majority (15/23, 65.2%) resulting in hydrocephalus (73.9%), ventriculitis, and periventricular edema (7/23, 30.4%). Cystectomy was accomplished in 60.9%, usually by removal of a fourth ventricular cyst through a suboccipital craniotomy. Nonresectable cysts were treated medically. Ventriculoperitoneal shunts were inserted in 43.5% (10/23) and failed in four, three from infection. Other complications included surgically induced injuries (4/23, 17.4%) and entrapment of a lateral ventricle (2/23, 8.7%). Despite a common severe early course, 90.9% (20/22) stabilized without recurrence, 15% (3/20) complained of mild-to-moderate neurological complaints, and 15% (3/20) were significantly disabled. Four patients who underwent removal of ventricular cysts without significant other NCC and who received with no cysticidal treatment became CSF cestode antigen negative without recurrence indicating that after successful extraction of cysts, additional cysticidal treatment may not be needed.

## INTRODUCTION

Neurocysticercosis (NCC) is caused by infection of the brain by larval cyst of the tapeworm *Taenia solium*. Clinical manifestations are variable, dictated by the parasite burden, location of the cysts, presence and degree of parasite degradation, and associated level of inflammation. Cysts located in the parenchymal, subarachnoid, and ventricular compartments of the brain tend to give rise to distinct clinical presentations that require specific surgical and/or medical approaches and therapies. Seizures and epilepsy are the most common manifestation of cysts lodged within the brain parenchyma. By contrast, cysts in the subarachnoid space give rise to symptoms related to mass effects and chronic arachnoiditis resulting in infarcts, nerve entrapments, and hydrocephalus.^1^ Manifestations of ventricular cysts are primarily because of complete, partial, or transient obstruction of cerebrospinal fluid (CSF) flow caused by obstructing or migrating cysts and associated ventriculitis.^2–4^ Unattached cysts may cause acute or episodic symptoms from transient obstruction, sometimes from head movement (Bruns syndrome).^5^ Degenerating cysts are commonly attached to the ependymal lining of the ventricles frequently causing acute or chronic ventriculitis,^6,7^ partial or complete obstruction of CSF flow, hydrocephalus, periventricular inflammation, and in the most severe form, a locked-in ventricle leading to herniation, if untreated.^8,9^

There are no randomized trials comparing management of ventricular disease, which includes emergent control of acute high-pressure hydrocephalus followed by extirpation of cysts through craniotomy or endoscopic extraction,^10,11^ shunt placement, or other drainage procedures, cysticidal treatment, and anti-inflammatory agents, commonly corticosteroids. Most reported series concentrate on the clinical presentation, initial treatments, and short-term clinical course. Less well discussed are the complications of ventricular disease itself including the treatment and outcome of retained ventricular cysts and surgical interventions and the long-term clinical course and sequelae. Here, we review our experience of ventricular NCC focusing on complications, prognosis, and long-term sequelae, as well as laboratory correlates of treatment success.

## METHODS

This series is a retrospective review of the medical records of all patients diagnosed with NCC with involvement of the ventricular system of the brain followed at the Clinical Center of National Institutes of Health (NIH) between March 1985 and October 2017. National Institutes of Health is a unique institution that only accepts referred patients usually diagnosed at other institutions. All signed the protocol consent and were enrolled into protocol NIH 85-I-0127 (a natural history protocol allowing evaluation, treatment, and follow-up of patients with NCC) approved by the IRB.

Inclusion required a diagnosis of NCC^12^ and the presence of one of more cysts within the brain ventricular system. These patients are a subset of 121 subjects enrolled and diagnosed with NCC. All patients were referred to NIH either before or after acute interventions and initiation of medical therapies at other centers. Treatment decisions at NIH were based on each patient’s medical state and the presence and stage of other forms of NCC. The latter commonly dictated the initial and/or long-term therapies. Patients were followed appropriately according to their medical status, but once active treatment was no longer required, stable patients were followed at the Clinical Center of the NIH minimally every 6 months and then annually indefinitely. Lumbar punctures and CSF analyses were initially not included as part of the routine serial testing but more recently was felt to add to the clinical decision making and included whenever possible.

Approaches to patient care, procedures, treatments, and testing changed over time and were dependent on a number of factors including the prevailing best treatment recommendations, presence of other forms of NCC, prior complications and treatments, available cysticidal medications, and expertise and ability of staff to perform endoscopic interventions.

Measurement of cestode antigen (Ag) followed a published method^13^ with some modifications (S. Mahanty, unpublished data). Briefly, a standard curve was generated using known quantities *T. solium* (Ts) cyst extracts, TsAg (10–3000 ng/mL in serial dilutions) as Ags on each plate of samples analyzed by enzyme-linked immunosorbent assay. The optical densities of test samples were interpolated on the standard curves to assign standardized values for TsAg (ng/mL). Reagents were supplied by Pierre Dorny (Department of Biomedical Sciences, Prince Leopold Institute of Tropical Medicine, Antwerp, Belgium). Cerebrospinal fluid samples were not treated with trichloroacetic acid (TCA), a technique used to dissociate Ag-antibody complexes when antibody excess occurs in a test solution, because pilot experiments to optimize the assay failed to show significant differences in Ag levels in the same samples analyzed with and without TCA treatment (data not shown). A known positive and six infection-negative samples from donors from a nonendemic region (USA) were included as controls with each test, and the cutoff values (in ng/mL) were determined to be the mean plus two standard deviations of the six negative control samples. This approach allowed comparison of Ag levels between assays run on different plates and on different days.

### Instructive case.

**Figure 1.**
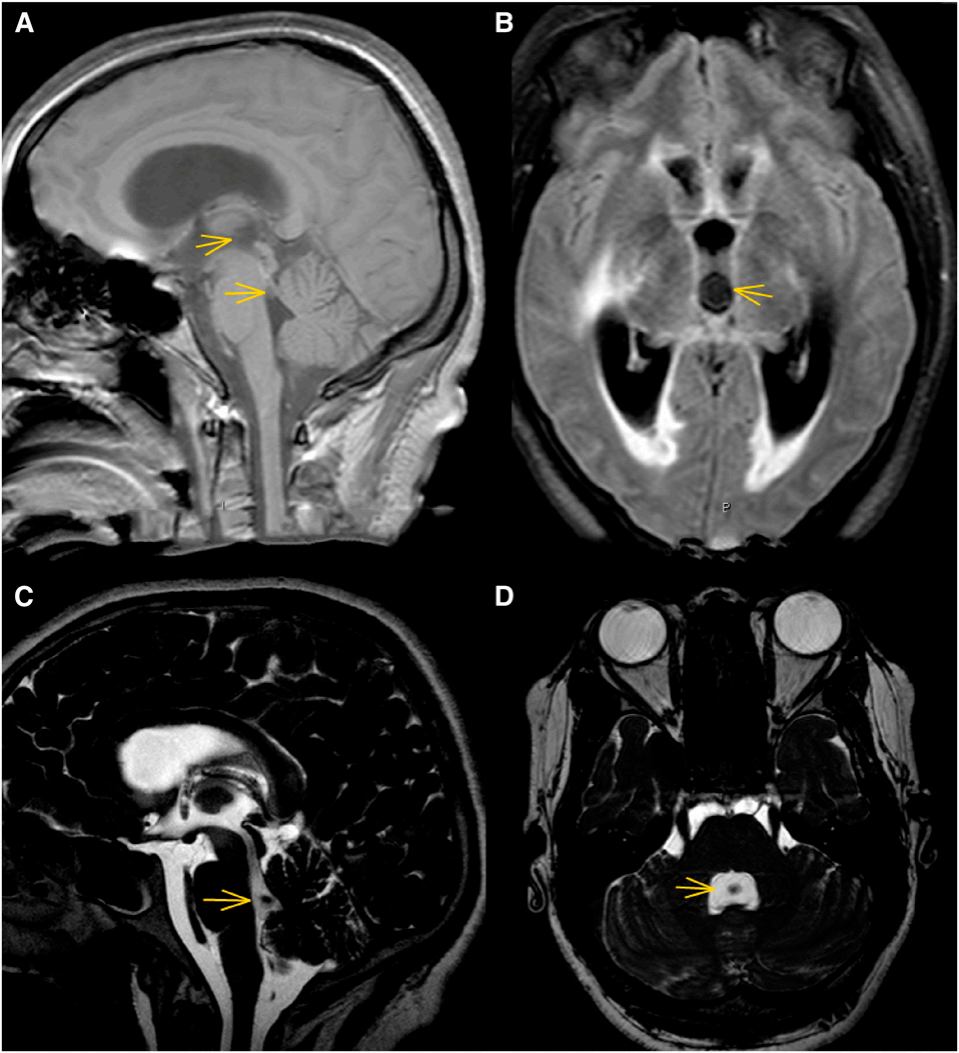
MRI imaging of the case. Panels (**A**) and (**B**) are imaging performed on the day of presentation, February 7, 2011, panel (**C**) on the day after presentation, February 8, 2011 and panel (**D**) on the day of admission to National Institutes of Health on June 2, 2011. Panel (**A**) is a sagittal T1 weighted fast field Echo (FFE) image revealing a barely visible third ventricle cyst showing [better seen in the axial view in Panel (**B**)] and part of the cyst in the process exiting the aqueduct into the fourth ventricle. Arrows delineate the cyst in the third and fourth ventricles. Panel (**B**) is an axial fast-attenuated inversion recovery image showing acute hydrocephalus with transependymal flow and a third ventricular cyst (arrow). Panel (**C**) is a cerebrospinal fluid-driven equilibrium radiofrequency reset pulse image demonstrating the scolex now fully situated in the fourth ventricle. Panel (**D**) is an axial balanced FFE image on June 2, 2011 (balanced fast field echo) image showing a cyst occupying the fourth ventricle (arrow) with a calcified scolex seen as a void in the middle of the cyst. This figure appears in color at www.ajtmh.org.

The patient is a 23-year-old Hispanic female (#14) who presented to an outlying hospital with headache, abnormal behavior progressing to coma on February 7, 2011. Magnetic resonance imaging and computed tomography imaging was interpreted as showing hydrocephalus with obstruction at the level of the fourth ventricle; no other identifiable abnormalities or lesions were seen. An external ventricular drain (EVD) was placed with marked neurological improvement. Multiple lumbar punctures were performed, which showed a variable number of white blood cells (WBC) ranging from 6 to 195/mm^3^ with 6–8% eosinophils. Cerebrospinal fluid was positive for antibodies to *T. solium* on two occasions. Evaluation for other chronic causes of meningitis was unrevealing. Although the diagnosis of NCC was considered, it was rejected. The patient’s acute symptoms resolved. Over the ensuing 4 months, the patient improved clinically but was not entirely well. She complained of headaches which became constant, poor memory, and fatigue. The rapid onset of dizziness, unsteadiness, and difficulty walking prompted admission to the NIH on June 2, 2011. The physical and neurological examinations were normal except for a mildly abnormal Romberg test. Before her first admission on February 7, 2011, she related several episodes of brief loss of vision. Review of the initial MRI imaging revealed a third ventricular cyst transiting into the fourth ventricle. Part of the cyst had entered into the fourth ventricle resulting in acute obstruction at the level of the third ventricle and aqueduct causing hydrocephalus and transependymal flow of the proximal ventricles (Figure 1A and B). A repeat MRI on the following day (Figure 1C) revealed the cyst and scolex (a void within the cyst) now present within the fourth ventricle. On admission to NIH, the presence of a fourth ventricular cyst and mild hydrocephalus was confirmed (Figure 1D). The cyst was removed via a suboccipital craniotomy without complications; no cysticidal drugs were administered. Ventricular Ag at the time of surgery was low positive. Subsequently, the size of the ventricular system and CSF parameters normalized at 5 months postoperatively. On November 1, 2011, lumbar CSF showed WBC of two and no detectable Ag. The patient has done well 3 years postsurgery but continues to complain of mild memory impairment.

## RESULTS

### Characterization of patients.

Twenty three of 121 (19.0%) patients with NCC had one or more ventricular cysts (Table 1). One patient who was lost to follow-up shortly after presentation was excluded. Of the 23 patients, 60.9% were male and the median age at presentation was 31.8 years. Just more than 40% (10/23, 43.4%) were diagnosed with NCC and had cyst(s) extracted/fenestrated at the referral institution, whereas about one fifth (5/23, 21.7%) had cysts surgically removed at NIH. All patients had continuing care at NIH including cysticidal treatment, corticosteroids, and other anti-inflammatory therapy as clinically needed. Patients were followed up for a median of 3.6 years (range: 0.1–30.5 years). The median time from leaving residence in an endemic country to presentation was 6.5 years (range: 0.17–16 years); one person who had continued yearly exposure was excluded and for two patients, the information was not available.

**Table 1:** Characterization and demographics of patients

Number of patients*	23/121 (19.0%)
Median age at presentation (years)	31.8 (range: 22.4–52.6)
Median duration of symptoms (years)	1.0 (range: 0–10.4)
Median years followed	3.6 (range: 0.1–30.5)
Median years postimmigration†	6.5 (*N* = 20, range: 0.17–16)
% Male	60.9%
% Other forms of NCC present	17/23 (73.9%)
% No cysticidal Rx after extraction	4/14 (28.6%)

NCC = neurocysticercosis.

* One patient seen once was excluded from analysis.

† Excluded one patient who returned to endemic regions yearly and the time of immigration was unavailable in two.

### Presence of other forms of NCC.

In addition to ventricular disease, other forms of NCC were also present in 17 of 23 patients (73.9%; Tables 1 and 2) and frequently dictated the need and type of cysticidal treatment. Subarachnoid disease (in 8/23, 34.8%) and calcifications (in 9/23, 39.1%) were the most common nonventricular forms of NCC present; viable parenchymal cysts were found in 5/23 (21.7%; Table 2). Only 6/23 (26.1%) of the patients had ventricular NCC as their sole manifestation; of these, four had a single fourth ventricular cyst, one had a single third ventricular cyst, and one had a lateral cyst and a small cyst in the aqueduct of Sylvius.

**Table 2:** Additional neurocysticercosis involvement

Other NCC involvement	Number of persons (%)
None	6 (26.1)
Subarachnoid	5 (21.7)
Calcification	4 (17.4)
Viable cyst, calcification	3 (13.0)
Viable cyst	2 (8.7)
Subarachnoid, calcification	1 (4.3)
Subarachnoid, spinal	1 (4.3)
Subarachnoid, spinal, calcification	1 (4.3)
Total persons with other involvement	17/23 (73.9)

### Location of cysts within the ventricles.

The location of the cysts within the ventricles is summarized in Table 3. The fourth ventricle was the most common site involving 16/30 (53.3%) of cysts in 15/23 (65.2%) persons. In three of these patients, cysts migrated into the fourth ventricle shortly after presentation. Eleven of thirty (36.7%) lateral ventricular cysts were present in 8/23 (34.8%) persons. Two or 6.9% of the cysts in 2/23 (8.7%) persons were located in the third ventricle. Multiple cysts were common; one person had four cysts, two in the fourth ventricle and one in each of the lateral ventricles, and three persons each had two cysts in the lateral ventricles.

**Table 3:** Location of cysts

Location*	Number of persons (%)
Fourth	11 (47.8)
Lateral†	5 (21.7)
Third migrated to fourth	2 (8.7)
Third migrated to fourth + lateral	1 (4.3)
2 cysts in fourth + 2 lateral	1 (4.3)
Third	2 (8.7)
Aqueduct + lateral	1 (4.3)
Location of individual cysts (%)	
Total fourth‡	16 (53.3)
Total lateral	11 (36.7)
Total third	2 (6.7)
Aqueduct	1 (3.3)

* Thirty total cysts.

† One lateral cyst migrated between anterior and posterior horns.

‡ Cysts that ended up in the fourth ventricle.

### Symptoms at presentation.

Symptoms at presentation ranged from none to severe impairment or coma (Table 4). Almost all the symptoms were because of acute or chronic obstruction resulting in hydrocephalus, which occurred in 17/23 (73.9%) of the patients. Symptoms included headache, nausea, vomiting, dizziness, and confusion, as well as acute onset of syncope or coma. Patient 14 had symptoms related to head movement and presented with acute onset of unconsciousness with a cyst obstructing the anterior fourth ventricle.

**Table 4:** Symptoms/signs at presentation

Symptom	Number of patients (%)
Hydrocephalus	17/23 (73.9)
Headache	14/23 (60.9)
Vomiting	8/23 (34.8)
Nausea	7/23 (30.4)
Syncope	6/23 (26.1)
No symptoms	4/23 (17.4)
Confusion/mentation	3/23 (13.0)
Vision abnormalities	3/23 (13.0)
Dizziness	3/23 (13.0)
Coma	2/23 (8.7)
Fever	1/23 (4.3)

Cysts did not cause symptoms in 2/5 persons with only lateral cysts compared with 2/12 asymptomatic persons with only fourth ventricular cysts (Fisher’s exact test, *P* > 0.05). Symptoms in those with lateral ventricular cysts were because of entrapment with localized hydrocephalus requiring a shunt for decompression in one patient and an asymptomatic entrapment not requiring a shunt in the second patient. In the third patient, symptoms were because of migration of a single cyst between the frontal to posterior horns of the right lateral ventricle.

Among the four persons who were asymptomatic, two had lateral cysts that resolved with/or without treatment and two had fourth ventricular cysts. Of these, one had a nonobstructing small fourth ventricular granuloma detected after a seizure because of a degenerating right frontal cyst and the second demonstrated an asymptomatic viable fourth ventricular and a silent Sylvian fissure cyst detected after imaging to evaluate trauma sustained at work.

### Interventions and shunt insertions.

Extraction of ventricular cysts that can safely be removed is currently the recommended treatment of choice for obstructing and most nonobstructing cysts (Table 5). In the present series, cysts were surgically removed in 14/23 (60.9%) of patients; 11/23 (47.8%) by way of a craniotomy to remove fourth ventricular cysts and 3/23 (12.5%) by endoscopy. One large third ventricular cyst, obstructing flow through the foramina of Monroe was decompressed by fenestration. Nine of 23 (39.1%) cysts, including the fenestrated cyst, were not removed and five persons required a shunt. Altogether, obstruction and/or hydrocephalus were controlled with ventriculoperitoneal shunts in 10/23 (43.5%) persons and in one patient with a ventriculostomy by way of an endoscopy. Shunts failed in 4/10 (40.0%) patients, three from secondary infection, which required one or more shunt replacements.

**Table 5:** Surgical interventions and shunt insertions

Interventions	Number of patients (%)
Cyst removed	14/23 (60.9)
Craniotomy	11/23 (47.8)
Endoscopy	3/23 (13.0)
Cyst retained*	9/23 (39.1)
Cyst fenestration	1/23 (4.3)
Shunt placed	10/23 (43.5)
Ventriculostomy	1/23 (4.3)
Shunt infection	3/10 (30.0)
Shunt failure	4/10 (40.0)

* One person had both retained lateral cysts that degenerated on treatment and two surgically removed fourth ventricular cysts.

### Complications.

**Figure 2.**
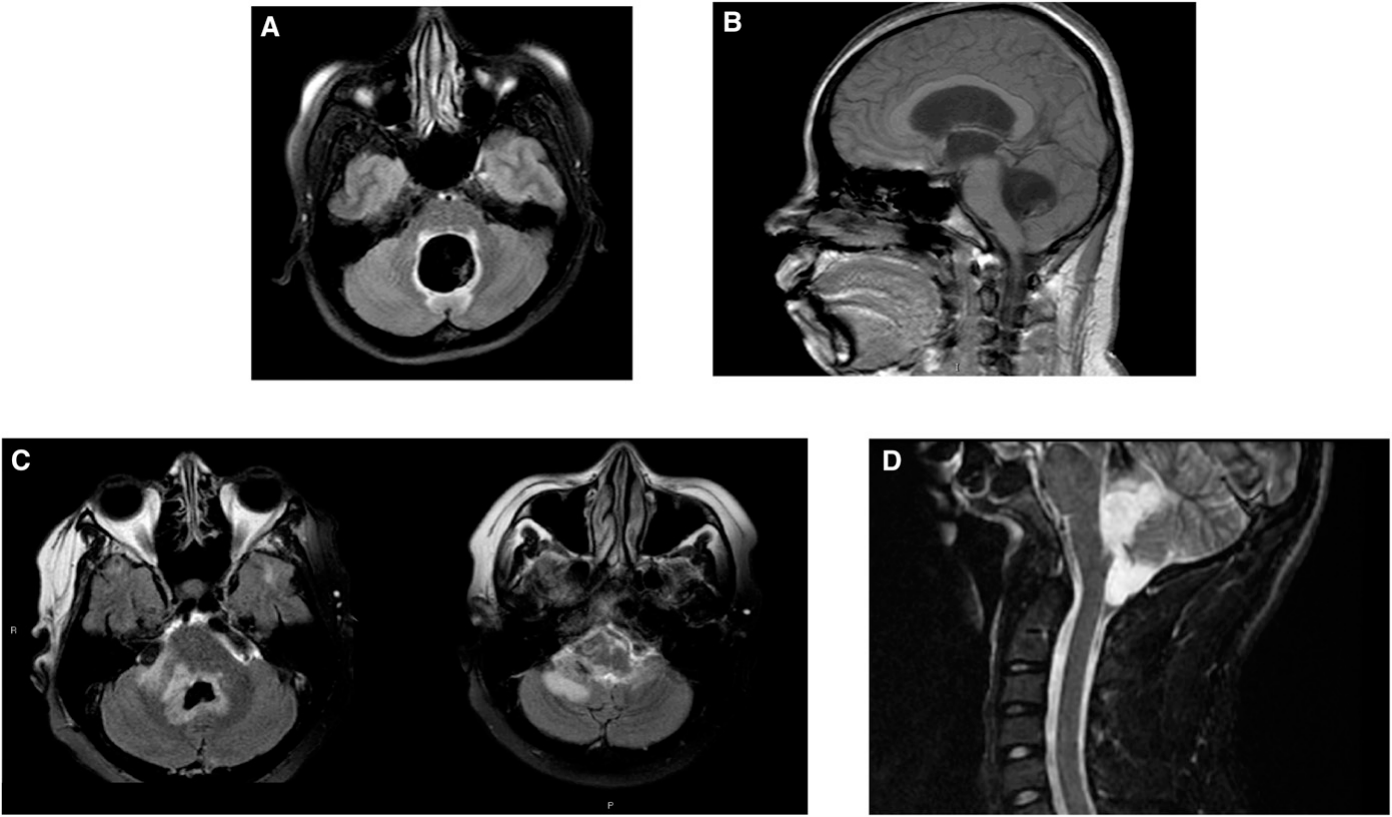
Panels (**A**) and (**B**) are Axial fast-attenuated inversion recovery (FLAIR) and sagittal T1W fast field Echo (FFE) imaging, respectively, of a patient with massive enlargement of the fourth ventricle with a scolex within the cyst. Hydrocephalus is apparent in the sagittal image. Panel (**C**) shows two FLAIR images demonstrating extensive periventricular edema around the fourth ventricle. Panel (**D**) is a lateral sagittal short-T1 inversion recovery image showing the fourth ventricle cyst that has exited the fourth ventricle into the cisterna magna by way of the foramen of Luschka.

Ventriculitis and periventricular edema, because of host reaction to residual fourth ventricular cysts, was the most common complication occurring in 7/23 patients (30.4%; Figure 2, Table 6). Unexpectedly, in some patients ventriculitis and edema resolved and then reoccurred, sometimes multiple times. Symptoms, mostly related to vision and extraocular movement, were because of involvement of contiguous structures surrounding the fourth ventricle and aqueduct of Sylvius. These patients often required high dose and long-term corticosteroids and/or other anti-inflammatory drugs. Similarly, entrapped fourth ventricular cysts occurred in three patients (13%). Compensated hydrocephalus, not requiring a shunt, occurred in two patients (8.7%). Endoscopy-related complications occurred in two patients. One person demonstrated a linear injury originating from the ventricular surface of the medulla and another bled into the lateral ventricle presumably from attempts to remove a cyst within the aqueduct migrating from the third to the fourth ventricle. Seizures from parenchymal disease, unrelated to ventricular cysts, also occurred in five patients (21.7%).

**Table 6:** Complications

Complications	Number of patients (%)
Periventricular edema	7 (30.4)
Residual fourth ventricle cyst	6 (26.1)
Seizure (parenchymal cyst)	5 (21.7)
None	5 (21.7)
Surgically induced bled	4 (17.4)
Entrapment (lateral ventricle)	2 (8.7)
Compensated hydrocephalus	2 (8.7)
Distal shunt abscess resulting in jejunal resection	1 (4.3)

### Clinical state at last evaluation.

All but two patients were followed up for at least 1 year, most considerably longer (Table 1). One patient was followed up for 6 months and although asymptomatic when last evaluated, because follow-up was limited, he was excluded from analysis. The other patient was clinically stable as judged by multiple visits over 7 months and therefore was included. Most patients were clinically stable without active disease or recurrent growth of subarachnoid cysts at the time of last assessment (20/22, 90.9%) and a majority or 11/20 (55%) of these were asymptomatic (Table 7). Once stabilized, none had regrowth or recurrence of disease or symptoms because of ventricular cysts. A few complained of mild-to-moderate symptoms related to prior ventricular or surgical interventions (3/20, 15.0%) or manifested significant disability (3/20, 15.0%). Two other patients experienced episodes of perilesional edema around calcifications,^14^ which were unrelated to ventricular disease. The observation period was too short to assess for recurrence in two patients.

**Table 7:** Clinical Status at last evaluation

Clinical status	Proportion (%)
Stable with no evidence of recurrence/progression*	20/22 (90.9) Stable with no evidence recurrence
	−11/20 (55.0) No complaints
	−3/20 (15.0) Significant disability
	−3/20 (15.0) Mild-mod complaints
	−2/20 (10.0) Episodes of perilesional edema
Short observation†	2/22 (9.1) Mild symptoms

NCC = neurocysticercosis.

* Excluded one patient who was lost to follow-up before 6 months.

† Less than 1 year treatment or extraction.

The three chronically disabled patients presented acutely requiring emergent surgical procedures. Complications and severe sequelae arose from the effects of the severe presentation itself and surgical interventions to remove cysts, shunt placements and replacements, and infections. Patient three presented with a greatly enlarged fourth ventricle caused by a degenerating cyst along with marked ventriculitis and periventricular inflammation and edema. The cyst was removed via a craniotomy. Bleeding within the ventricle, which was filled with debris, was noted postoperatively. Hydrocephalus did not resolve and a shunt was therefore inserted, which was revised but ultimately another shunt was required. The patient developed cerebellar encephalomalacia resulting in gait abnormalities and dizziness that improved slowly. Patient 10 presented with a cyst in transit from the third to fourth ventricle, totally occluding CSF flow. The cyst was extracted endoscopically but complicated by ventricular bleeding and injury to the aqueduct. The patient was unable to communicate or walk but eventually recovered both activities with rehabilitation. However, remaining sequelae included difficulty in cognition and incapacitating short-term memory deficit. Patient 17 suffered from chronic headaches, which escalated to severe persistent headaches, confusion, and nausea and vomiting 2 days before admission because of a posterior third ventricular cyst. An EVD was placed and he was initially treated with cysticidal drugs without corticosteroids but developed near herniation. The patient had a complicated hospital course in which he developed two episodes of bacterial ventriculitis requiring multiple cycles of EVD insertions and antibiotic treatment. Ventricular bleeding was documented. After stabilization, the cyst was removed while receiving high dose corticosteroids. A functioning shunt was eventually placed successfully. Long-term sequelae consisted of headaches, debilitating short-term memory loss, and depression.

### Radiological and laboratory course of patients with only ventricular disease.

A subset of six patients with fourth ventricular cysts and a single patient with a third ventricular cyst with either no other NCC lesions (patients 1, 10, 14, and 17) or with additional parenchymal involvement (patients 9 and 19 with only a calcification) were monitored with one or more lumbar punctures (Table 8). All showed resolution of ventriculitis by imaging, normalization, or CSF pleocytosis and negative or low CSF cestode Ag. The four persons who had their cysts extracted and who had no other NCC lesions improved in the absence of cysticidal treatment, indicating cyst removal alone was sufficient treatment.

**Table 8:** Final CSF evaluation of clinically stable patients with ventricular involvement and no other forms of NCC or only parenchymal NCC

Patient*	Cysticidal treatment	Other NCC	LP	CSF WBC†	Cestode Ag†
1	Yes	No	Yes	3	Neg
9	No	C	Yes	3	Neg
10	No	No	Yes	1	Neg
14	No	No	Yes	2	Neg
17	No	No	Yes	3	Neg
19	Yes	C	Yes	1	Very low

Ag = antigen; C = calcification; CSF = cerebrospinal fluid; LP = lumbar puncture; NCC = neurocysticercosis; Neg = negative; WBC = white blood count.

* Either only a third (patient 17) or a fourth ventricular cyst (patients 1, 9, 10, 14, and 19) or with parenchymal calcification.

† Final LP WBC and cestode Ag.

### Cestode CSF Ag results.

One or more lumbar CSF samples were available for cestode Ag testing in 15/23 patients (65.2%), irrespective of presence of other forms of NCC. Of these, 10 had detectable Ag initially but subsequently no detectable Ag in lumbar CSF; none of these patients developed clinical or radiological disease but two with concomitant subarachnoid NCC have not been followed up for more than a year following the end of cysticidal treatment. Of 5/16 who had a single Ag CSF determination (four with low but detectable levels, one during treatment, and one after treatment), none has experienced disease recurrence. In the remaining eight patients, who did undergo lumbar punctures, none has developed clinical or radiological relapse/recurrence. All but one has been followed up for greater than a year.

## DISCUSSION

The clinical presentation of ventricular NCC is relatively specific because symptoms are primarily because of acute and/or chronic obstruction of CSF flow and associated inflammation, usually centered in and around the fourth ventricle. The present series is similar to other reports^2,4,15–18^ with regard to proportion of NCC with ventricular involvement, clinical presentation and, in more recent series, proportion of patients undergoing surgical cyst removal. Other forms of NCC frequently occurred in the presence of ventricular disease; parenchymal calcifications was most common other involvement followed by subarachnoid disease, similar to the high occurrence noted by Mexican investigators.^17^

The incubation period of ventricular disease appears to be considerably longer than parenchymal disease. Almost all our patients had no or limited travel back to endemic areas after immigration to the United States and documented acquired infection in the United States is rare. Patients were diagnosed a median of 6.5 years after immigration. If we assume no significant exposure after immigration and a lifetime of exposure before immigration, then infection near the time of immigration seems improbable and the incubation period is likely 10 years or greater. In comparison, the incubation of British soldiers who returned from India and who were subsequently diagnosed primarily with parenchymal NCC had a mean incubation period from the midpoint of residence in India to presentation of 4.8 years.^19^ This compares to a mean in our patients of 7.0 years from the time of arrival in the United States to diagnosis.

A number of our patients suffered from severe complications including ventriculitis, periventricular inflammation and edema, locked-in ventricles, and lateral ventricle entrapment. Although these are reported complications, they are usually not delineated in published series making it difficult to know whether they are more or less frequent compared with our series. The most serious and acutely ill patients had symptoms related to retained, enlarged, and inflamed cysts, consistent with prior experience highlighting that the presence of gadolinium enhancement of fourth ventricular cysts (indicative of inflammation) was significantly more likely to require reoperation.^6,7^ Inflamed cysts caused mass effect, enlargement, and obstruction of the foramina of Luschka and Magendie^8,9^ and less commonly the foramina of Monroe.^20^ In cysts lodged in the proximal fourth ventricle obstructing the outflow of the aqueduct of Sylvius, inflammation sometimes extended into the aqueduct, at times resulting in signs and symptoms related to dysfunction of the adjacent nuclei and pathways controlling eye movements. Recurrence of inflammation after apparent resolution was a feature of some retained fourth ventricular cysts. In a few instances, rebound inflammation was provoked by abrupt and/or premature cessation of corticosteroid treatment, similar to what has been seen in other types of NCC. Lateral ventricular entrapment leading to localized hydrocephalus is another complication barely mentioned in published series but occurred in two of our patients. The possibility of developing entrapments is a good reason to consider surgical removal of lateral cysts. However, a majority of unremoved cysts located in the lateral ventricles resolved without sequelae after treatment.

There are no randomized or controlled studies of treatment of ventricular NCC, which is therefore based on expert consensus and individual experience. However, over the prior decades, investigators observed a clinical benefit of surgical removal of cysts.^4,21^ When the less invasive technique of endoscopic removal became available, a general consensus evolved to remove cysts that can be excised safely. The rational for extraction is to remove cysts because they act as reservoirs of released Ag that drives the host’s inflammatory response leading to ventriculitis, fibrosis, and hydrocephalus. There is an increasing number of studies demonstrating the utility and safety of endoscopy.^10,11,22,23^ In many but not in all situations, endoscopy appears to be a less morbid procedure to explore the ventricular system, remove intraventricular cysts, and to perform intraventricular CSF diversionary procedures such as ventriculostomy and septotomy^21^ to control hydrocephalus and avoid the need for external shunting. Although endoscopic surgery has many advantages compared with craniotomy, the procedure can cause severe complications and intraventricular hemorrhage. However, removal of fourth ventricular cysts through the aqueduct of Sylvius can damage the aqueduct leading to significant morbidity; a posterior occipital craniotomy may be a safer approach, particularly in those with less experience.^24^ Placement of shunts is still necessary to control hydrocephalus when internal drainage or cyst removal is not possible. Both shunt malfunction and infections are well-described morbid complications. Besides the presence of retained cysts, repeated surgeries and shunt replacements with associated episodes of hydrocephalus and surgical complications were sources of considerable morbidity in this series.

Our data suggest that simple removal of cysts may avoid the need for further cysticidal treatment. In the six patients who had primarily ventricular disease and whose cysts were removed, all had normalization of MRIs and all those with analyzable CSFs demonstrated normalization of pleocytosis and loss or a low cestode Ag levels (median 170 days, range: 61–238 days from NIH admission). Four of these patients did not receive cysticidal treatment postextirpation, indicating treatment may not be required following cyst removal.^2^ One of our patients who had two fourth ventricular cysts removed subsequently developed compensated hydrocephalus emphasizing that hydrocephalus can develop after cyst removal,^10^ reinforcing the need for close follow-up after cyst extraction. Corticosteroids are commonly used to control ventriculitis and possibly to prevent development of hydrocephalus but its utility is unstudied in this context. In contrast to our experience, Khade et al.^25^ analyzed the literature and reported patients who had cysticidal treatment after cyst removal developed hydrocephalus less frequently. Unfortunately, variables that likely affect the development of hydrocephalus, including the duration, dose, indication for use of corticosteroids, or the presence of concomitant subarachnoid disease, was neither mentioned nor evaluated.

Others have measured cestode Ag levels in patients with combined subarachnoid and ventricular or subarachnoid and parenchymal involvement and showed decreased cestode Ag levels in a subset who developed damaged cysts.^17^ These results suggest that a decrease in the concentration of cestode Ag indicates effective treatment of combined subarachnoid and ventricular cysts and logically for either subarachnoid or ventricular disease when present alone. However, because no clinical information was included in this report, it is unclear whether a decrease in CSF Ag levels predicts clinical benefit. In our series, lack, loss, or low levels of cestode Ag in the lumbar CSF in those whose CFS values were due to ventricular cysts and who did not receive cysticidal treatment following cyst removal predicted clinical benefit without recurrence. The latter suggests cysticidal treatment is not required after ventricular cyst extraction.

The long-term outcome of ventricular disease is infrequently mentioned and usually not well documented.^7^ Fifteen of the 22 persons were followed up long enough to assess the presence and extent of disability related to ventricular cysts and clinically stable posttreatment over varying lengths of time. The cohort was followed a median of 3.6 years (range: 0.1–30.5 years). Of these, three were acutely ill at presentation and likely would have died without immediate intervention, which, unfortunately, resulted in complications and significant disabilities including gait disturbances, slow movement, and poor memory. As noted earlier, the increase in disability was mostly related to retained cysts and complications from multiple surgical interventions including hemorrhage, penetration injury to the brain, multiple shunt insertions and reinsertions, and shunt infections. Four patients complained of recent memory impairment apparently related to the development of acute hydrocephalus. Symptoms related to other NCC involvements were also common.

Our approach to the treatment of ventricular disease evolved over the time period of this series. Early on, based on a medically treated series by Proano et al.,^26^ patients were treated with anti-parasitic drugs and corticosteroids and/or methotrexate^27^ to control inflammation over the long-term. With ability to remove cysts endoscopically or otherwise, cyst removal became our preferred approach followed by cysticidal therapy and anti-inflammatory agents. Later, we documented that cysticidal treatment was not necessarily required following removal of obstructing cysts. However, the series is small and recurring hydrocephalus because of continuing ventriculitis and scarring is still possible although at this point unlikely in those followed for years.^10^ Retained cysts are treated with long-term cysticidal treatment and anti-inflammatory agents and serially monitored with repeat lumbar punctures, MRI imaging, and CSF analyses including cestode Ag levels. Normalization of CSF parameters, lack of detectable cestode Ag, and improved MRI imaging have been indicative of nonrecurrence and clinical stabilization over time.

This study has several drawbacks. Accrual of patients occurred over an extended period of time and therefore procedures and treatments changed. In addition, most patients were referred to the NIH, usually following necessary surgical procedures and initiation of medical treatment, both of which varied depending on the hospital, as well as knowledge and practice of caring physicians. On the other hand, at the NIH, patients were followed closely and extensively evaluated during treatment and their course documented by annual visits usually for extended periods of time. This group is among the most detailed and longest followed up cohort of patients with ventricular NCC to date.

This study describes the treatment and follow-up of well-evaluated patients followed up for an extended time. The overall prognosis is good for most; but a minority suffers from significant morbidity.
